# Autoimmune Hemolytic Anemia Exacerbation Associated With COVID-19 Infection and Markedly Elevated Inflammatory Markers

**DOI:** 10.7759/cureus.20416

**Published:** 2021-12-14

**Authors:** Tyler A Finkenthal, Zackery Aldaher, Salman Ahmed, Louis DiValentin

**Affiliations:** 1 Internal Medicine, Alabama College of Osteopathic Medicine, Dothan, USA; 2 Internal Medicine, Regional Medical Center, Anniston, USA

**Keywords:** inflammatory mediators, inflammatory marker, sars-cov-2 (severe acute respiratory syndrome coronavirus -2), acute exacerbation, cytokine storms, warm autoimmune hemolytic anemia, coronavirus disease 2019 (covid-19), autoimmune hemolytic anemia (aiha)

## Abstract

The association between previously diagnosed autoimmune hemolytic anemia and exacerbations due to coronavirus disease 2019 (COVID-19) infection is a rare phenomenon that is not well understood. In this case, we present a 68-year-old female with a past medical history significant for systemic lupus erythematosus (SLE), splenectomy, and autoimmune hemolytic anemia (AIHA) since childhood that had been very well controlled with only one previous exacerbation. This patient’s chief complaint and clinical symptoms at admission were related to hemolytic anemia and not active COVID-19 infection. This case report reveals a possible association between the hyperinflammatory syndrome caused by COVID-19 and the exacerbation of previously well-controlled autoimmune diseases.

## Introduction

Warm autoimmune hemolytic anemia (WAHA) leads to accelerated red blood cell destruction caused by the reaction of autoantibodies with red blood cells [1]. It can be a chronic relapsing disease with a reported incidence of one to three cases per 100,000 and is clinically characterized by anemia, reticulocytosis, elevated indirect bilirubin levels, low haptoglobin, and a positive direct antiglobulin test in 95% of cases. While 50% of primary WAHA cases are idiopathic, a variety of disorders can elicit an increased risk of secondary WAHA including rheumatic diseases like systemic lupus erythematosus (SLE) [2].

Coronavirus disease 2019 (COVID-19) is an infectious disease that is asymptomatic in some cases; however, severe disease can be complicated by pneumonia, respiratory failure, and acute respiratory distress syndrome [3,4]. Evidence now increasingly supports that severe cases of COVID-19 are associated with a hyperinflammatory syndrome and cytokine release [5]. A hyperinflammatory syndrome characterized by fulminant hypercytokinemia with multiorgan failure has already been linked to COVID-19 disease severity [6].

Seven cases of autoimmune hemolytic anemia (AIHA) have been reported as a complication of a COVID-19 infection with anemia symptoms beginning within the timeframe associated with hypercytokinemia, noted as four to 13 days after symptom onset [5]. In addition, a case of a rapidly exacerbating AIHA with markedly elevated cytokines has been reported in a case of non-COVID-19 pneumonia [7].

This case report describes a 68-year-old female with a history of WAHA secondary to SLE that was well controlled. She presented with symptoms of anemia and jaundice following a recently diagnosed COVID-19 infection. Using this case study as an example, the hyperinflammatory state caused by COVID-19 has the potential to exacerbate well-controlled autoimmune diseases as seen in this patient.

## Case presentation

A 68-year-old Caucasian female with a history of recurrent AIHA since childhood, splenectomy, and systemic lupus erythematosus presented to the emergency department with severe generalized weakness for the past week. Nine days prior, the patient was diagnosed with COVID-19 at a local urgent care after being evaluated for a sore throat, loss of smell and taste, cough with green sputum production, and rhinorrhea. She received one dose of intramuscular steroids and was sent home with a course of azithromycin which she had completed. After this diagnosis, the patient became increasingly weak and developed aching suprapubic abdominal pain which she rated at a five out of 10 in severity. On presentation, she reported her cough had improved, and sore throat and rhinorrhea had resolved, but loss of smell and taste were still present. She also endorsed being jaundiced but denied fevers, chills, sweats, rashes, dyspnea, chest pain, lightheadedness, syncope, dizziness, nausea, vomiting, diarrhea, or urinary symptoms. She has a past medical and surgical history that also includes prediabetes mellitus, gastroesophageal reflux disease, fibromyalgia, and appendectomy. The patient’s medications include calcium carbonate cholecalciferol 600 mg daily, clonazepam 0.5 mg every night, folic acid 1 mg every day, isosorbide mononitrate extended release 30 mg every morning, methocarbamol 750 mg every four hours as needed, and nitroglycerin 0.4 mg every five minutes as needed. The patient was not vaccinated against the COVID-19 virus and had not previously required treatment for her AIHA in the past four years. However, she did display evidence of low-grade hemolysis including an elevated reticulocyte count, lactate dehydrogenase (LDH), and indirect bilirubin with a hemoglobin between 10 and 11 gm/dL. 

Physical examination revealed a well-developed and nourished female with jaundice and mild tenderness in the suprapubic region on palpation. Mucous membranes were moist, pupils were equally round and reactive to light, extraocular movements were intact, and neck was supple with normal range of motion and no lymphadenopathy. On further examination, lungs were clear to auscultation bilaterally, heart was regular rate and rhythm with no abnormal heart sounds, skin was warm and dry, abdominal sounds were present and normoactive, and abdomen was soft. The patient was alert and oriented to person, place, and time; cranial nerves II-XIII were intact; and she had a normal finger to nose exam and exhibited no lateralizing deficits. 

At the time of admission, chest x-ray showed no acute process. Table 1 shows the results of her complete blood count (CBC) with differential at the time of admission. Table 2 reveals the results of her complete metabolic panel (CMP) at the time of admission, and Table 3 reveals the results of further laboratory studies performed at time of admission. WAHA was diagnosed given a characteristic laboratory profile that included anemia, elevated reticulocyte count with macrocytosis, low haptoglobin, elevated LDH, and direct antiglobulin test (DAT) results. 

**Table 1:** CBC With Differential at Time of Admission CBC: complete blood count, WBC: white blood cells, RBC: red blood cells, MCV: mean corpuscular volume, RDW: red blood cell distribution width, MCHC: mean corpuscular hemoglobin concentration, MCH: mean corpuscular hemoglobin.

CBC With Differential at Admission	Patient’s Results	Normal Range
WBC	13.3	4.5-10.4 x 10^3/μL
RBC	1.41	3.70-5.30 x 10^6/μL
Hemoglobin	7.4 gm/dL	11.0-16.0 gm/dL
Hematocrit	20.1%	35.0%-47.0%
MCV	142.6 fL	81.0-97.0 fL
RDW	24.4%	12.1%-16.2%
MCHC	36.8 gm/dL	32.5-36.1 gm/dL
MCH	52.5 pg	27.7-33.3 pg
Reticulocyte count	18.6%	0.5%-1.5%

**Table 2:** CMP at Time of Admission CMP: complete metabolic panel, AST: aspartate aminotransferase, ALT: alanine aminotransferase, ALP: alkaline phosphatase.

Complete Metabolic Panel at Admission	Patient’s Results	Normal Range
Potassium	5.3 mmol/L	3.5-5.1 mmol/L
Albumin	3.3 gm/dL	3.4-5.0 gm/dL
Total bilirubin	5.2 mg/dL	0.20-1.00 mg/dL
Direct bilirubin	1.3 mg/dL	0.00-0.20 mg/dL
AST	85 unit/L	2-33 unit/L
ALT	33 unit/L	13-61 unit/L
ALP	145 unit/L	45-117 unit/L

**Table 3:** Further Laboratory Studies at Time of Admission dsDNA: double-stranded DNA, TIBC: total iron-binding capacity, CRP: C-reactive protein, LDH: lactate dehydrogenase, SARS-CoV-2: severe acute respiratory syndrome coronavirus 2, PCR: polymerase chain reaction.

Further Laboratory Studies at Admission	Patient’s Results	Normal Range
Urine urobilinogen	2.0 E.U./dL	<2.0 E.U./dL
dsDNA	10,425 IU/mL	Positive >10 IU/mL
Direct Coombs test	IgG and C3D positive	
Iron	231 mcg/dL	50-170 mcg/dL
TIBC	246 mcg/dL	250-450 mcg/dL
CRP	6.64 mg/L	0.00-3.00 mg/L
Ferritin	1278.6 ng/mL	8.0-252.0 ng/mL
LDH	886	84-246
Haptoglobin	<31.0 mg/dL	30.0-200.0 mg/dL
COVID-19 rapid antigen test	Negative	Negative
SARS-CoV-2 PCR	Positive	Negative

The patient was admitted to the hospital and started on 1 mg of folic acid daily and 1 mg/kg dose of prednisone daily with plan to taper and transfuse if her hemoglobin level reached below 7.0 gm/dL. The patient’s respiratory status was monitored during her inpatient stay, and she remained stable with an oxygen saturation of 96%-100% on room air. Hemoglobin level continued to drop and, on the third day of her stay, reached a level of 5.7 gm/dL (normal: 11.0-16.0 gm/dL). Hematology was consulted, and the patient received three total units of least incompatible packed red blood cells (RBCs) and a dose of intravenous rituximab. Patient’s hemoglobin eventually rose to 14.0 gm/dL (normal: 11.0-16.0 gm/dL) and hematocrit rose to 46.1% (normal: 35.0%-47.0%) after a six-day inpatient stay, indicating a remarkably quick improvement. Patient was discharged home on 40 mg of oral prednisone daily with taper and one dose of rituximab for three weeks. She was scheduled for outpatient follow-up with hematology, and she has been stable since. 

## Discussion

Autoimmune hemolytic anemia (AIHA) is an autoimmune disorder characterized by hemolysis and consequent activation of the complement pathway that can present with anemia and acrocyanosis [5]. Warm autoimmune hemolytic anemia (WAHA) is a subtype of AIHA along with cold agglutinin syndrome and paroxysmal cold hemoglobinuria [8]. If the clinical presentation suggests WAHA, then confirmatory testing with a direct antiglobulin test can be administered to determine positivity for IgG only or for IgG ± C3d to red blood cells [9]. Infection, autoimmune disorders, and hematologic malignancies are known triggers for AIHA; this case illustrates that infection with COVID-19 may be a possible trigger as well. AIHA affects about one to three per 100,000 people a year with about 70%-80% of the cases being WAHA [8]. WAHA can affect patients of any age, but it is more common in 50-70 year olds, with a median age of onset at 52 years. It is more common in women than in men. The association between COVID-19 infection and first-time onset of WAHA has been reported [5].

The proposed pathophysiology of AIHA includes autoimmune antibodies that react with antigens on the surface of the red blood cell (RBC) membrane. Binding of these autoantibodies results in recognition and phagocytosis by splenic macrophages in a process called extravascular hemolysis [8]. Considering postsplenectomy status of this patient, it is important to note further pathophysiologic mechanisms of AIHA including direct complement-mediated lysis occurring primarily in the circulation as well as in the liver [10]. Common secondary triggers of WAHA are autoimmune disorders, hematologic malignancies, and viral and bacterial infections (parvovirus B19, hepatotropic virus, human immunodeficiency virus (HIV), mycoplasma pneumonia, mycobacterium tuberculosis, brucella, and syphilis). The likely mechanism by which viral infections may induce AIHA is via molecular mimicry to antigenic epitopes on RBC proteins or carbohydrates [10]. The complement system, autoantibodies, phagocytes, cytokines, and CD8+ and CD4+ T cells all play an important role in secondary WAHA [8]. In the case of WAHA and complement activation, mainly the IgG3 antibody subclass binds to erythrocytes and activates the classical complement system through fixation of the complement protein C1. However, IgM may also contribute but dissociates at warmer, more central parts of the body [10]. At 37 degrees, warm IgG antibodies will bind to the cell surface antigens on the RBCs and will be phagocytosed mainly by splenic macrophages which contain Fc-gamma receptors that recognize IgG heavy chains resulting in extravascular hemolysis [8]. 

Anemia characterized by lower levels of hemoglobin at admission has been shown to be significantly associated with more severe COVID-19 infections measured by longer length of hospital stay and mortality [11]. The most common hematologic autoimmune disorders associated with COVID-19 infection in a collection of studies were immune thrombocytopenic purpura and then AIHA [12]. There were seven cases from French and Belgian hospitals of first episode AIHA with a median time of onset from COVID-19 infection to AIHA of nine days [5]. Another case reported COVID-19 triggered AIHA in a patient with a history of oropharyngeal carcinoma which is not known to be a trigger for AIHA [3]. 

Molecular mimicry is a possible underlying mechanism of COVID-19 causing hemolytic anemia [13]. Ankyrin-1 (Ank-1), an erythrocyte membrane protein which connects the RBC skeleton to the plasma membrane and is important for RBC function and differentiation, is commonly defective in patients with hereditary spherocytosis and has been identified to have an immunogenic-antigenic epitope with 100% identity to the spike glycoprotein of severe acute respiratory syndrome coronavirus 2 (SARS-CoV-2) [13]. It is known that peptides of COVID-19 share similarity to peptides of alveolar surfactant protein resulting in tissue damage through the process of molecular mimicry and subsequent pulmonary disease. Furthermore, COVID-19 shares similar proteins to those found in the human body such as interleukin 7 which predisposes to severe lymphopenia and histone-lysine N-methyl transferase C which is linked to neurodevelopmental and behavioral abnormalities [14]. Therefore, molecular mimicry is one plausible theory as to why COVID-19 can exacerbate or initiate AIHA.

Previously diagnosed cases of AIHA exacerbated by COVID-19 infection have yet to be reported. This case is unique in that it demonstrates reactivation of a previously latent AIHA by a COVID-19 infection with elevated inflammatory markers such as ferritin and C-reactive protein. Prior case reports indicate an association with COVID-19 infection and new-onset AIHA as well as AIHA exacerbation in non-COVID pneumonia with hypercytokinemia [5,7]. This case highlights the importance of understanding the pathophysiology of the cytokine storm associated with COVID-19 infection in worsening a previously well-controlled autoimmune disease. Molecular mimicry may also play a role. Further research is needed to better understand the relationship between COVID-19 and AIHA. Furthermore, the ability of COVID-19 to cause worsening symptoms of previously diagnosed, well-controlled autoimmune diseases must be further explored. In future case reports, an emphasis on the patient’s inflammatory parameters at admission and the degree of exacerbation in clinical symptoms associated with previously diagnosed autoimmune disease will be important to note. 

## Conclusions

The coexistence of a previously diagnosed autoimmune hemolytic anemia being exacerbated by an active COVID-19 infection has not been reported to our research or knowledge. This case provides further support in a relationship between the hyperinflammatory state caused by COVID-19 and worsening of a patient’s previously well-controlled autoimmune disease. Additionally, it calls for further research into the possibility of latent autoimmune disease reactivation secondary to COVID-19 infection, noting the patient’s inflammatory markers at clinical presentation.
